# The Effect of Inclusion Criteria on the Functional Properties Reported in Mouse Visual Cortex

**DOI:** 10.1523/ENEURO.0188-20.2021

**Published:** 2021-02-23

**Authors:** Natalia Mesa, Jack Waters, Saskia E. J. de Vries

**Affiliations:** 1 Allen Institute, Seattle, WA 98109; 2 University of Washington, Seattle, WA 98195

**Keywords:** calcium imaging, data analysis, inclusion criteria, neurophysiology, visual cortex

## Abstract

Neurophysiology studies require the use of inclusion criteria to identify neurons responsive to the experimental stimuli. Five recent studies used calcium imaging to measure the preferred tuning properties of layer 2/3 pyramidal neurons in mouse visual areas. These five studies employed different inclusion criteria and reported different, sometimes conflicting results. Here, we examine how different inclusion criteria can impact reported tuning properties, modifying inclusion criteria to select different subpopulations from the same dataset of almost 17,000 layer 2/3 neurons from the Allen Brain Observatory. The choice of inclusion criteria greatly affected the mean tuning properties of the resulting subpopulations; indeed, the differences in mean tuning because of inclusion criteria were often of comparable magnitude to the differences between studies. In particular, the mean preferred temporal frequencies (TFs) of visual areas changed markedly with inclusion criteria, such that the rank ordering of visual areas based on their TF preferences changed with the percentage of neurons included. It has been suggested that differences in TF tuning support a hierarchy of mouse visual areas. These results demonstrate that our understanding of the functional organization of the mouse visual cortex obtained from previous experiments critically depends on the inclusion criteria used.

## Significance Statement

Inclusion criteria are widely used in physiological studies to limit analysis to active or responsive neurons, yet the impact of the criteria employed on the ensuing analyses are rarely considered. We have compared the effect of several inclusion criteria used in published studies comparing visual responses across cortical visual areas in the mouse cortex by applying these to a single dataset. The choice of inclusion criteria greatly affected the mean tuning properties of the resulting subpopulations; indeed, the differences in mean tuning because of inclusion criteria were often of comparable magnitude to the differences between studies.

## Introduction

**Figure 1.**
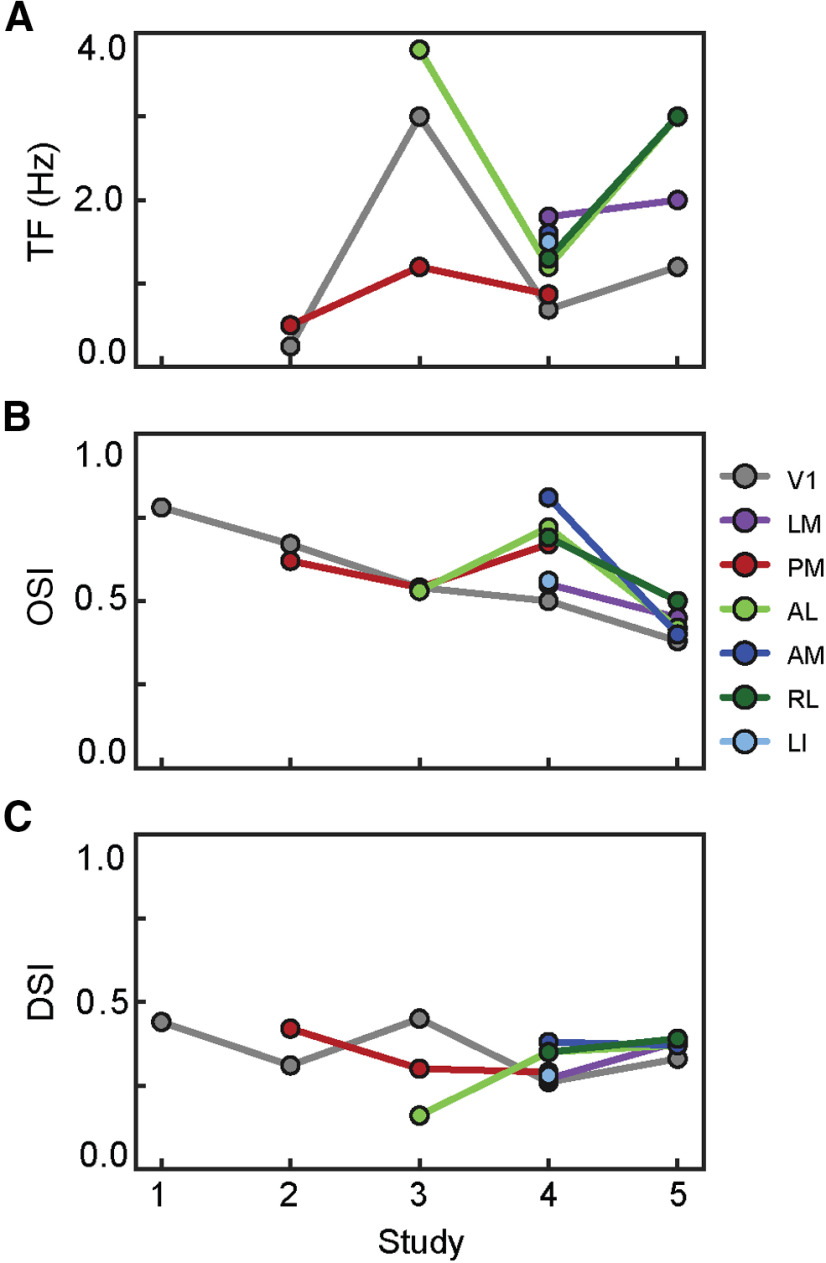
Tuning characteristics in published studies. ***A***, Mean preferred TF tuning of seven visual areas reported in five published studies. ***B***, ***C***, Same as in ***A*** but reporting the OSI and the DSI.

Five recent studies have employed two-photon calcium imaging to compare spatial frequency (SF) tuning, temporal frequency (TF) tuning, orientation selectivity, and directional selectivity of neurons across mouse visual cortical areas (Table 1; Fig. 1; Andermann et al., 2011; Marshel et al., 2011; Roth et al., 2012; Tohmi et al., 2014; Sun et al., 2016). Some results were consistent across studies, e.g., the mean preferred TF of neurons in area AL was greater than those in V1 (Fig. 1*A*), but there were also differences between studies, e.g., some studies found that the mean preferred TF of neurons in PM was greater than those in V1 while others found the opposite. Further, the magnitudes of average TF tuning, orientation selectivity index (OSI), and direction selectivity index (DSI) in individual visual areas as well as the rank order of these properties between visual areas differed across studies (Fig. 1). All five studies imaged layer 2/3 of mouse visual cortex and activity was evoked with a drifting grating stimulus, but the studies differed in anesthesia state, calcium indicator, stimulus parameters, and in the inclusion criteria used in analysis (Table 1). It is likely that all these differences contribute to the contrasting results. Here, we leverage a single large and open dataset, the Allen Brain Observatory, to quantify the impact of the choice of inclusion criteria on the measurement of tuning properties of neurons in mouse visual areas.

**Table 1:** Summary of the experimental conditions and inclusion criteria used in published studies

Paper	Anesthesia	Indicator	Stimulus	Stimulus repetitions	Responsiveness criteria	Percentage of responsive cells	# Cells Allen Brain Observatory
Study 1 Sun et al. (2016)	None	GCaMP6s	12-s full-field square grating TF: 0.5, 1 Hz SF 0.05cpd 8–16 directions	10	Mean ΔF/F > 10%	49% (*n* = 1279/2609)	17.5% (*n* = 2962/16923)
Study 2 Roth et al. (2012)	Urethane	OGB-1	5-s full-field sine wave grating TF: 0.5, 1, 2, 4 Hz SF: 0.01, 0.02, 0.04, 0.08, 0.16cpd 8 directions	4	In 50% of trials, mean ΔF/F >baseline + 3σ Mean response > 5%	44% (*n* = 399/973)	6.3% (*n* = 1068/16923)
Study 3 Andermann et al. (2011)	None	GCaMP3	40° sine wave grating patches TF: 0.5, 1, 2, 4, 8, 15, 24 Hz SF: 0.02, 0.04, 0.08, 0.16, 0.32cpd Direction: upward	9–28	*t* test comparing grating response with blank sweep with Bonferroni correction (*p* < 0.05/n)	8% (*n* = 28/340)	29.6% (*n* = 5015/16923)
Study 4 Marshel et al. (2011)	Isofluorane	OGB-1	4-s full-field sine wave gratings SF: 0.04 cpd TF: 0.5,1, 2, 4, 8 Hz 8 directions	5	Mean ΔF/F > 6% Reliability >1(see Materials and Methods)	42% (*n* = 586/1395)	7.8% (*n* = 1068/16923)
Study 5 Tohmi et al. (2014)	Urethane	Fura-2	5-s ramping square and sine wave gratings SF 0.05 and 0.1cpd 8 directions	28–124	Max ΔF/F > 5%	41.2% (*n* = 142/347)	94.7 (*n* = 16,033/16,923)

Last column shows the number of neurons selected from the Allen Brain Observatory.

**Figure 2.**
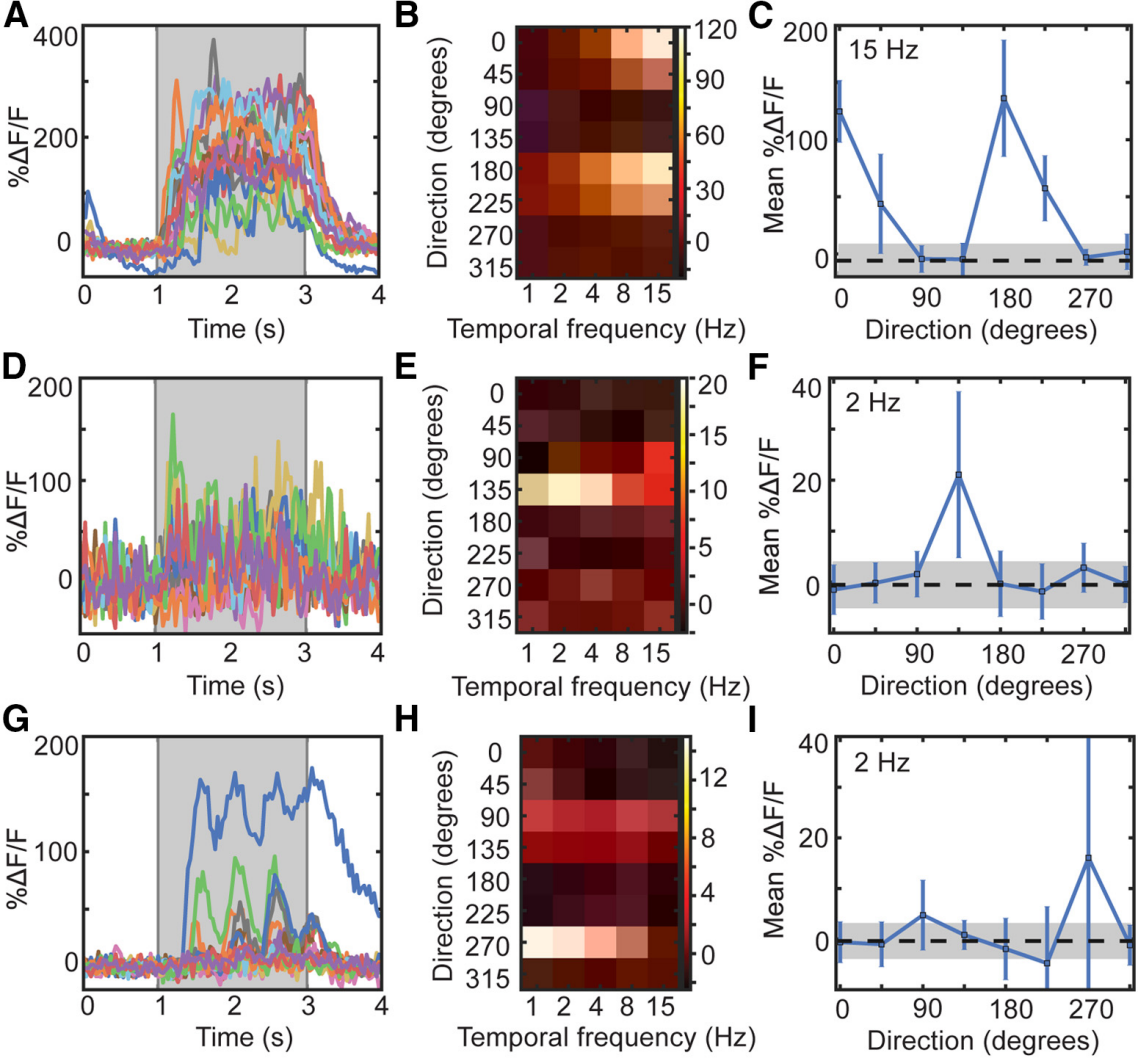
Example cells that pass inclusion criteria exclusively. ***A***, All DF/F responses to the preferred stimulus condition (TF and direction) of a cell that passes all published inclusion criteria. ***B***, Heatmap of mean %DF/F responses to each stimulus condition (TF × direction). ***C***, Mean %DF/F responses (± SEM) to stimuli of different grating directions in the same example cell. ***D–F***, Same as in ***A*** but with a cell that passes most criteria, but not Study 2. ***G–I***, Same as in ***A*** but with a cell that only passes Study 1 criteria.

Calcium imaging studies usually require the use of inclusion criteria to select neurons that are deemed to be “active” or “responsive” such that the derived analysis of their activity is relevant to the aims of the experiment and not a quantification of noise. As the measured fluorescence shows continuous fluctuations, these criteria serve to identify which fluctuations reflect signal rather than noise. Criteria are often based on the amplitude of the fluorescence change, e.g., a threshold on the mean or median change in fluorescence over multiple trials, or its reproducibility, e.g., a statistically significant stimulus-evoked change in fluorescence on a subset of trials. Naturally, some neurons exhibit large-amplitude changes in fluorescence on every trial in response to a preferred stimulus and fulfil both amplitude and reproducibility criteria (Fig. 2*A–C*). Many neurons display reproducible, small-amplitude changes (Fig. 2*D–F*) or large-amplitude changes in fluorescence on only some trials (Fig. 2*G–I*). Although not often used as the basis for inclusion criteria, other features of the fluorescence traces, such as periodicity in the fluorescence in response to a periodic stimulus such as a drifting grating (Fig. 2*I*) and tuning to stimulus characteristics such as orientation and TF (Fig. 2*C*,*H*,*I*), may also be suggestive of stimulus-evoked activity (Niell and Stryker, 2008).

Each of the five studies used different inclusion criteria and it is unclear whether these different criteria select the same or different neurons and how they impact the distribution of measured responses to visual stimuli across the population. Here, we explore the effects of inclusion criteria on results from a single large dataset, eliminating the effects of different experimental conditions. We used recordings from the Allen Brain Observatory, a database of physiological activity in visual cortex measured with two-photon calcium imaging from adult GCaMP6f transgenic mice (de Vries et al., 2020). We found that tuning properties varied with inclusion criteria, in some cases changing the rank order of tuning properties across mouse cortical visual areas.

## Materials and Methods

### Stimulus and dataset

We used calcium imaging recordings from the Allen Brain Observatory, a publicly available dataset that surveys physiological activity in the mouse visual cortex (de Vries et al., 2020). We specifically used the responses to the drifting grating stimulus in this dataset. This stimulus consisted of a 2 s grating followed by a 1s mean luminance gray period. Six TFs (1, 2, 4, 8, 15 Hz), eight different directions, and one SF (0.04 cpd) were used. Each grating condition was presented 15 times.

Data analysis was performed in Python using the AllenSDK. The evoked response was defined as the mean dF/F during the 2-s grating presentation. Responses to all 15 stimulus presentations were averaged together to calculate the mean evoked response.

We restricted our analysis to cells in layer 2/3 (175–250 μm below pia) of transgenics lines Cux2-CreERT2;Camk2a-tTa;Ai93 and Slc17a7-IRES2-Cre;Camk2a-tTa;Ai93, which express GCaMP6f in neural populations in layer 2/3 and throughout neocortex, respectively. A total of *N* = 16,923 neurons from 66 mice (42 male, 24 female) were used for this analysis.

### Metrics

The preferred direction and TF condition was defined as the grating condition that evoked the largest mean response. In order to compute the average TF tuning of a population of neurons, these TF values were first converted an octave scale (base 2), averaged, then converted back to a linear scale and reported.

Direction selectivity was computed for each neuron as the following:

$$DSI=\frac{R_{pref}-R_{null}}{R_{pref}+R_{null}},$$

where $R_{pref}$ is the mean response at to the preferred direction and $R_{null}$ is the mean response to the opposite direction.

Orientation selectivity was computed for each neuron using the global OSI (OSI; Ringach et al., 1997), defined as the following:

$$OSI=\frac{\sum^{}R_{\theta }e^{2i\theta }}{\sum^{}R_{\theta }},$$

where $R_{\theta }$ is the mean response at each orientation *θ*.

The coefficient of variance (CV) was used as our metric to determine robustness. CV was calculated for each neuron as the ratio of SD of the 15 responses to the preferred condition (mean dF/F over the 2-s stimulus presentation) to the mean evoked response (see above). A low CV would indicate high robustness.

Metrics were either computed using all available trials, or with cross validation. When using cross validation, half of the trials (chosen at random, without replacement) were used to identify the preferred direction and TF, and the other half of the trials were used to compute the metrics using those preferred conditions. This was iterated 50 times, and the resulting metrics were averaged together.

When examining the effects of the number of trials, for each number of trials (*n*), *n* trials were chosen at random (without replacement), and the cross-validation was done as described above.

### Inclusion criteria

Published studies used the following inclusion criteria, which we applied to cells in the Allen Brain Observatory dataset in the following manner:

Study 1: The mean evoked response (dF/F) to the preferred stimulus condition is >10% (Sun et al., 2016).

Study 2: In 50% of trials, the response is (1) larger than the 3× the SD of the prestimulus baseline and (2) larger than 5% dF/F (Roth et al., 2012).

Study 3: Paired *t* test (*p* < 0.05) with Bonferroni correction comparing the mean evoked response during the blank sweeps with mean evoked responses to preferred stimulus condition (Andermann et al., 2011).

Study 4: (1) The mean response (dF/F) to any stimulus condition is is >6%. And (2) reliability >1 where:

$reliability=\frac{R_{pref}-R_{blank}}{\sigma_{pref}+\sigma_{blank}}$ (Marshel et al., 2011).

Study 5: The maximum fluorescence change (dF/F) during the 2-s stimulus presentation block to any stimulus condition was >4% (Tohmi et al., 2014).

### Code availability

The code used in this paper is available at https://github.com/nataliamv2/inclusion_criteria.

## Results

**Figure 3.**
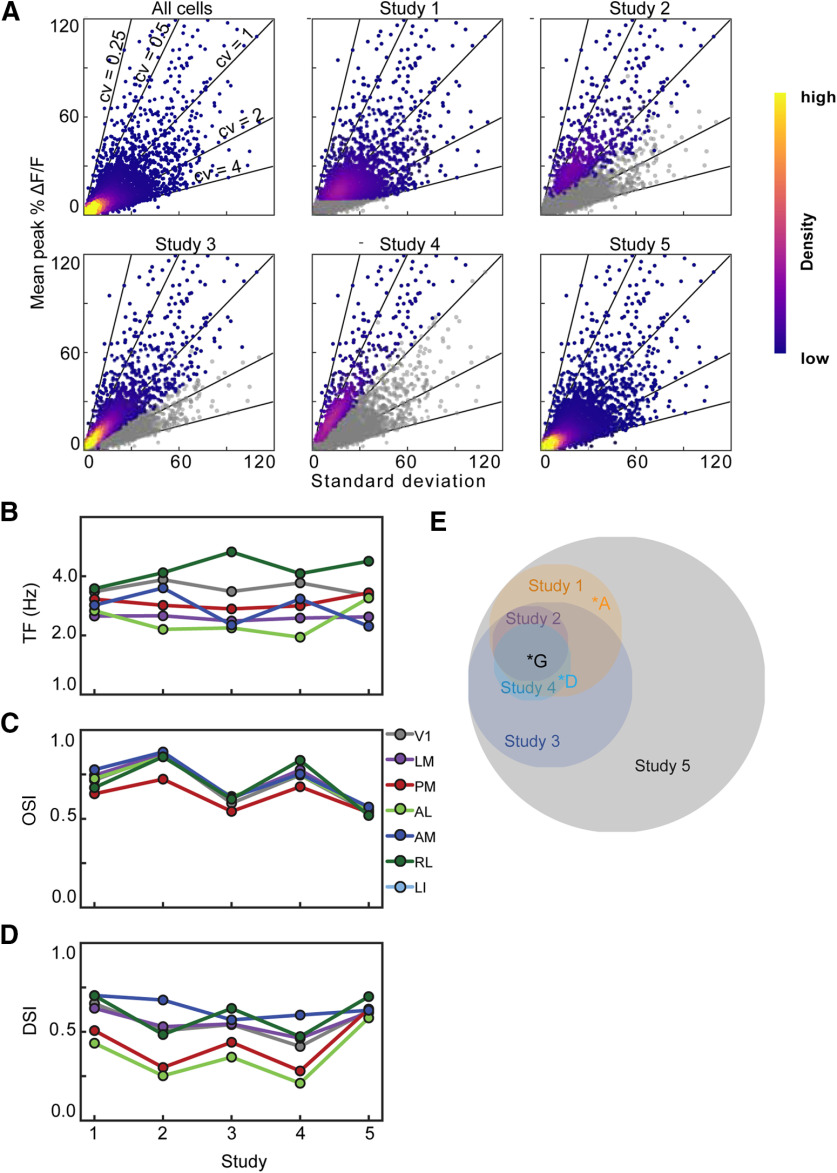
Most studies select for neurons along similar axes of the data. ***A***, Six density plots of the mean response at the preferred stimulus condition (%DF/F) against the SD of the responses at the preferred stimulus condition where each point represents a single neuron. For each study, colored neurons are those selected for by inclusion criteria. Heatmap represents the density of neurons. ***B–D***, Tuning characteristics after inclusion criteria are applied to Allen Brain Observatory. ***B***, Mean TF tuning of six visual areas when different inclusion criteria are applied. ***C***, ***D***, show the mean OSI and DSI of six visual areas, respectively. ***E***, Venn Diagram of neurons that were selected for by each inclusion criteria. Area of circles represents the number of neurons. Letters indicate example neurons from Figure 2.

The five studies employed a range of inclusion criteria, selecting 8–49% of the neurons in their respective studies (Table 1). The inclusion criteria were based on one or both of the amplitude and the trial-to-trial variability of the evoked responses and we therefore calculated the mean and SD of the response of each neuron to its peak stimulus condition (the direction and TF that evoked the largest mean response). We applied the five different inclusion criteria to the Allen Brain Observatory, a large two-photon calcium imaging data set. We restricted our analysis to layer 2/3 excitatory neurons imaged 175–250 μm below the pia in Cux2-CreERT2;Camk2a;Ai93 and Slc17a7-IRES2-Cre;Camk2a;Ai93 mice, yielding a dataset of fluorescence recordings from 16, 923 neurons. Different inclusion criteria selected different, often overlapping populations of neurons (6–94% of 16,923 neurons; Table 1, column 7), readily visualized by plotting the mean against the SD of the response (Fig. 3*A*). The results derived using these different criteria covered similar ranges to those in the published studies, consistent with the idea that effects of inclusion criteria could contribute to the disparate results across published studies (Fig. 3*B*).

**Figure 4.**
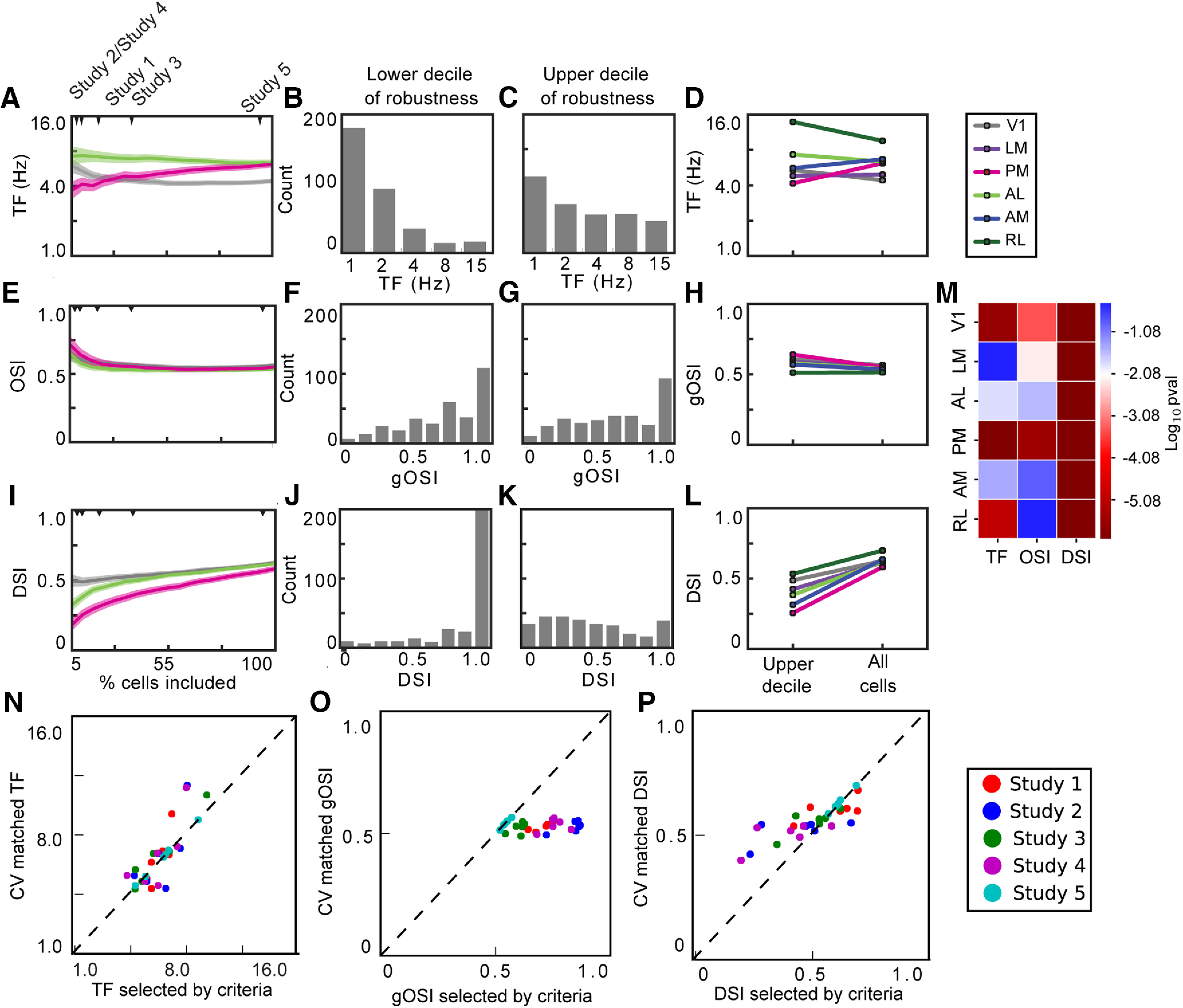
Tuning characteristics of neurons based on robustness. ***A***, ***E***, ***I***, Mean TF, OSI, and DSI tuning of neurons in V1, AL, and PM based on what percentage of most robust cells (cells with low CV) are included in the analysis. Shaded regions indicate SEM. The minimum percentage most robust cells displayed is 5%. ***B***, Distribution of TF tuning of 10% least robust cells. ***C***, Distribution of TF tuning of 10% most robust cells. ***F***, ***G***, ***J***, ***K***, Same as in ***B***, ***C*** but with OSI and DSI. ***D***, ***H***, ***L***, Mean TF, OSI, and DSI tuning of neurons in all visual areas comparing the 10% most robust neurons to the entire population of neurons. ***M***, Heat map displaying *p* values for Mann–Whitney *U* test comparing the 10% most robust neurons and the entire population of neurons. The color scale is centered at *p* = 0.05/6 to account for Bonferroni correction. ***N***, Mean DSI calculated for neurons selected to match the mean CV for each the neurons selected by each criterion, for each area, compared with the mean DSI for the neurons selected by that criteria and area. ***O***, ***P***, Same as in ***M*** but for OSI and TF.

Using CV (CV = SD/mean) as a measure of response robustness, we asked how increasing the number of neurons selected, from the most robust (lowest CV) to the least (highest CV), affects the computed tuning metrics. For some metrics, including more neurons affected tuning properties by almost as much as the differences between studies. For example, increasing included neurons changed the mean preferred TF for V1, PM, and AL as well as the rank order of these three areas, such that AL and PM display different mean TFs when only the top decile are included, but have the same mean TF when all neurons are included (Fig. 4*A–D*,*M*). Within V1, the change in mean TF reflects the fact that the highest decile (10% with highest CV) shows a broader distribution of preferred TF than the lowest decile (Fig. 4*B*,*C*). In contrast, the effect on on OSI was smaller and more consistent across areas, having a smaller effect on the value or the rank order across areas (Fig. 4*E–H*,*M*). Finally, increasing the number of neurons included increased the mean DSI, and did so consistently and significantly across all visual areas (Fig. 4*I–L*,*M*). The increase in DSI reflects the fact that many of the neurons in the lowest decile have a DSI of 1, whereas the neurons in the highest decile have a uniform distribution of DSIs (Fig. 4*J*,*K*).

None of the inclusion criteria used in the published studies apply a threshold on the CV specifically, but some incorporate measurements of reliability that might have a similar effect. If criteria are selecting neurons based primarily on reliability, one might expect that selecting a population of neurons with matched mean CV would result in similar tuning properties and would replicate the differences observed between the studies. We selected populations of neurons that had the same mean CV as those chosen by each inclusion criteria, for each area separately, and compared the tuning properties for that population to the tuning properties for the neurons chosen by the criteria. For some metrics, there was a high correlation between these values, namely mean preferred TF and mean DSI (*r* = 0.82 Pearson’s correlation for both; Fig. 4*N*,*P*). For preferred TF the values were close to unity, indicating that selecting neurons by their CV closely matched the differences between studies. For DSI, however, the range of DSI values was more limited. Thus, while there was a high correlation between the values for neurons selected by CV to those for neurons selected by the criteria, the shallow slope of this relationship made it less predictive. Further, for the mean OSI, the was no correlation between these values (*r* = 0.09; Fig. 4*O*). Thus, some of the differences between the published studies could result from the inclusion criteria effectively selecting neurons based on their reliability at different threshold. However, it is clear that the criteria did not select neurons exclusively based on the reliability, as captured by the CV, as CV alone cannot account for all of the differences between the studies.

Selection by CV displayed a greater effect on preferred TF and DSI than on OSI, likely because the measurements of preferred TF and DSI are more susceptible to noise. The neurons with the noisiest responses (greatest CV) commonly displayed DSI ∼1 (Fig. 4*J*), which is inevitable when the response to the null direction is 0. The response to the preferred direction need not be large and could even result from a single trial having just a small amplitude fluorescence change. As the preferred TF is the TF at which the neuron has its largest response, regardless of amplitude or reliability, the TF tuning is similarly sensitive to small numbers of noisy events. In contrast, OSI is calculated from the responses to all eight directions of drifting gratings and is thus less sensitive to a small amplitude response in one condition.

**Figure 5.**
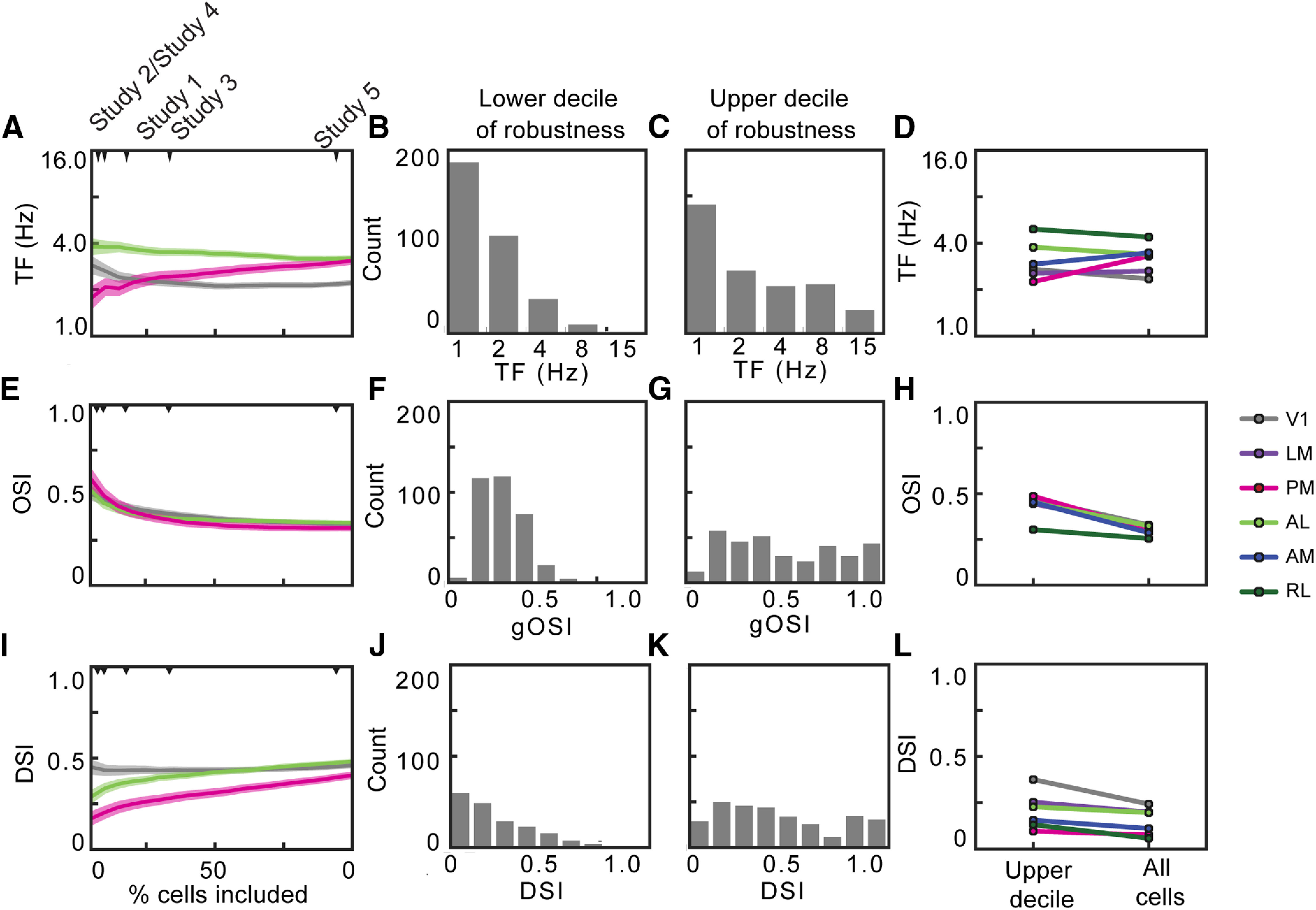
Tuning characteristics of neurons based on robustness with cross-validated metrics. ***A***, ***E***, ***I***, Mean TF, OSI, and DSI tuning of neurons in V1, AL, and PM based on what percentage of neurons are included in the analysis, starting with the most robust neurons. Shaded regions indicate SEM. The minimum percentage most robust cells displayed is 5%. ***B***, Distribution of TF tuning of 10% least robust neurons. ***C***, Distribution of TF tuning of 10% most robust neurons. ***F***, ***G***, ***J***, ***K***, Same as in ***B***, ***C*** but with OSI and DSI. ***D***, ***H***, ***L***, Mean TF, OSI, and DSI tuning of neurons in all visual areas in 10% most robust neurons versus the entire population of neurons.

Might a calculation that is more robust to trial-to-trial variability reduce the sensitivity of measurements to inclusion criteria or CV? We recalculated OSI, DSI and TF with cross-validation, using half of the trials to identify the stimulus condition that evoked the largest mean responses (grating direction and TF) and then calculated OSI, DSI and TF for these preferred conditions from the other half of the trials. The overall effect of including more neurons based on their CV on the cross-validated metrics across different areas was similar to that on the non-cross-validated metrics (Fig. 5). The notable difference is that the noisy neurons in the lowest decile of robustness no longer have high DSI or OSI values, but are shifted to much lower values (Fig. 5*F*,*J*). This difference is also reflected in the fact that the overall curves are shifted to lower values (compare Figs. 5*E*,*I* and Fig. 4*E*,*I*). Thus, while more statistically robust metrics calculated through cross-validation likely better reflect the true values of the population, they do not reduce the impact of selection on those metrics.

**Figure 6.**
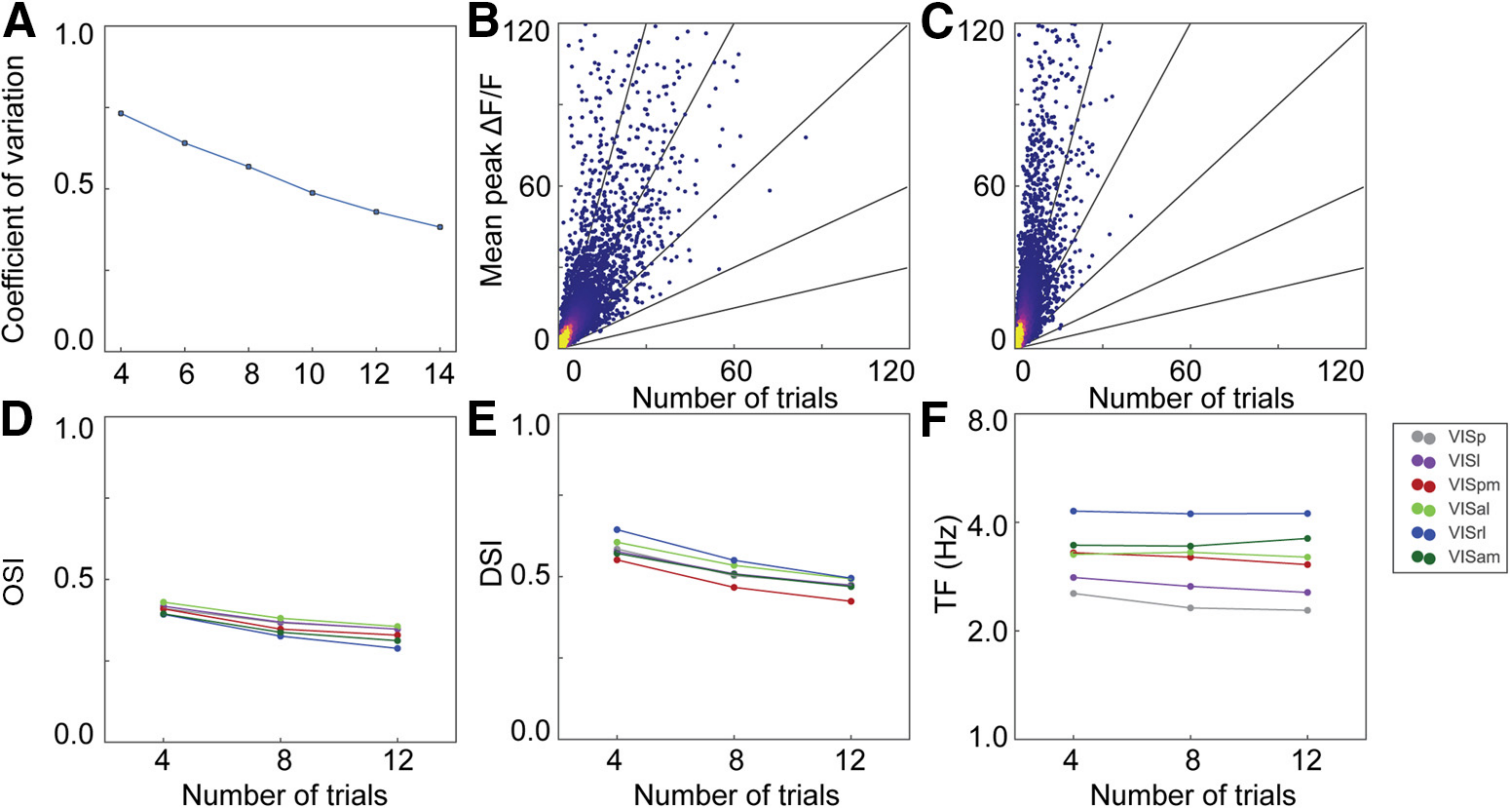
How trial number changes tuning metrics and CV. ***A***, Mean CV calculated at the preferred condition using different numbers of trials and the cross-validation method. ***B***, Mean peak response at the preferred condition versus SD at the preferred condition using only four trials and the cross validation method. ***C***, Same as in ***B*** but using 14 trials. ***D–F***, OSI, TF, and DSI calculated using the cross-validation method as a function of the number of trials used in the analysis.

Different studies presented each visual stimulus multiple times, with numbers of repetitions ranging from 4 to 24 trials (Table 1). Might the number of repetitions account for some of the differences between studies? We computed OSI, DSI and preferred TF using subsets of 4–14 trials. As expected, the variability of the responses decreased as the number of trials increased, resulting in a lower mean CV across the entire population (Fig. 6*A*). Visualizing the neurons by plotting response mean versus SD for *n* = 4 trials (Fig. 6*B*) and *n* = 14 trials (Fig. 6*C*), it is clear that the bulk of the data are shifted to more robust responses. Increasing the number of trials had a small effect on the cross-validated metrics (Fig. 6*D–F*), decreasing both the mean OSI and DSI across all areas (when including all neurons). The effect was consistent across all areas, however, thus the number of trials did not impact the rank order across areas. Thus, while more trials can reduce the variability of the response measurements, it is unlikely that these differences had a large effect on the differences observed between studies.

## Discussion

We applied different inclusion criteria to the Allen Brain Observatory two-photon dataset to examine how these criteria impact the reported tuning properties across visual areas after experimental differences are eliminated. That different inclusion criteria selected different subsets of neurons might not be surprising, but the extent of the differences between selected neurons was substantial. One key difference was in the numbers of neurons selected. To examine how including more, or fewer, neurons could impact the tuning properties, we used CV as a metric of robustness and shifted our threshold for inclusion. Mean TF, OSI, and DSI changed differently with the robustness of the responses of the underlying neurons. The preferred TF was the most sensitive, OSI the least sensitive.

Our results offer one possible explanation why published studies comparing TF, OSI, and DSI across mouse visual areas have produced different results for TF and more similar results for OSI and DSI. Mean TF tuning is more sensitive than OSI and DSI to the neurons selected. As a result, comparison across studies is difficult and there remains considerable uncertainty regarding the mean TF and the rank order of TF tuning across mouse visual areas.

We used CV to examine how including more neurons can impact the reported results, as one of the big differences of the criteria is the number of neurons they select from our dataset. But this is not the only difference between these criteria. The Venn diagram (Fig. 3*E*) reveals that the cells selected by different criteria are not described by a set of concentric circles, and neurons with mean CV matched to those selected by an inclusion criterion have different tuning (Fig. 4*O*,*P*), revealing that the inclusion criteria use features of the neural responses in addition to the size and reliability of neurons’ responses to their preferred condition. For instance, the statistical tests employed in Studies 3 and 4 also depend on the size and reliability of the neurons’ responses to the blank sweep.

Cross-validating metrics and increasing the number of trials can each improve the accuracy of the measured responses. Cross-validation can mitigate the impact of particularly noisy responses, reducing the impact of small numbers of outlier trials. This is most evident in the effect of cross-validation on the DSI distribution for the neurons in the lowest decile of robustness (Fig. 5*J*). It is possible that inclusion criteria based on the reliability of metrics across iterations of cross-validation might be more effective for identifying neurons with truly robust responses.

Our results illustrate how inclusion criteria can play a role in determining the tuning properties of visual areas. The choice of inclusion criteria is unlikely to account for all of the differences observed between the original studies, indicating that other experimental factors are important. Other factors likely include anesthesia state, the type of anesthesia used, the calcium indicator, image brightness, as well as visual stimulus parameters. Brain state can modulate neural responses in visual cortex, and anesthesia in particular can impact both the spontaneous and evoked responses. The type of anesthesia can also be a factor, with urethane impacting spontaneous and evoked firing rates but not OSI (Niell and Stryker, 2010) and atropine affecting OSI but not spontaneous firing rate, evoked firing rate, DSI, preferred TF, or preferred SF (Durand et al., 2016). Stimulus parameters, such as the size or contrast of the drifting gratings or the precise SFs and TFs, do also impact the evoked responses and could account for some of the differences observed between the original studies.

Calcium indicators have different sensitivities and signal-to-noise properties (Hendel et al., 2008; Chen et al., 2013), such that thresholds in mean DF/F appropriate for one indicator might not be appropriate for another. Most of the inclusion criteria selected ∼40–50% of neurons when applied to their own data, but when applied to the Allen Brain Observatory data the percentage of neuron included often differed substantially, presumably because experimental conditions such as indicator brightness differed across studies. For example, simple thresholds on peak DF/F cannot be applied uniformly across different calcium indicators. Thus, it is unlikely that a single set of inclusion criteria would be appropriate across a wide range of experimental conditions, and that these criteria must be chosen and validated by experimenters, including, for instance, an analysis of how metrics change based on how restrictive criteria are (Kim et al., 2018).

Functional specialization of the higher visual areas in mouse cortex has been interpreted as evidence of parallel streams (Andermann et al., 2011; Marshel et al., 2011). For example, V1 is thought to transfer low TF, high SF information to PM, the putative gateway to the dorsomedial stream (López-Aranda et al., 2009; Polack and Contreras, 2012; Glickfeld et al., 2013). However, in some studies, neurons in V1 and PM have similar mean TF tuning (with PM’s being 1.3–2× that of V1; Marshel et al., 2011; Roth et al., 2012), while others show that mean TF tuning in PM neurons that is 1/3 that of V1 neurons (Andermann et al., 2011). Our results indicate that in the most robust neurons, V1 has a higher TF tuning than PM, but in the least robust neurons, PM has a higher TF tuning than V1, potentially explaining the some of the difference between studies. Since TF is sensitive enough to inclusion criteria to change the relative order of TF tuning, it is difficult to interpret the relative TF tuning between visual areas currently. The most appropriate inclusion criteria would take into account how downstream targets filter or weight inputs and how robustness factors into that weighting. Since we do not know what this weighting is, we must be cautious in drawing conclusions about functional organization from these analyses.

## Synthesis

Reviewing Editor: Nicholas J. Priebe, University of Texas at Austin Decisions are customarily a result of the Reviewing Editor and the peer reviewers coming together and discussing their recommendations until a consensus is reached. When revisions are invited, a fact-based synthesis statement explaining their decision and outlining what is needed to prepare a revision will be listed below. The following reviewer(s) agreed to reveal their identity: Nathalie Rochefort, Marius Pachitariu.

Both reviewers highlighted a number of strengths of the work which demonstrates how inclusion inclusion criteria alter physiological estimates based on calcium signals in visual cortex. They also noted, however, weaknesses of the study. First, both reviewers agreed that while the manuscript focuses on inclusion criteria, it is also the case that the experimental paradigms varied across the 5 studies, and that the particular paradigms used also impacts our perspectives on the population of neuronal responses. Second, while the authors demonstrate that inclusion parameters alter differences in physiological estimates, they do not offer evidence that this ocurred in any of those studies. In addition to these comments, both reviewers provided detailed suggestions which you should find useful in revising your manuscript, if you decide to resubmit it to eNeuro or to another journal.

REVIEWER 1

ADVANCES THE FIELD

The main issue is that studies 1-5 not only differ in selection criteria, but also in a number of other experimental variables that could critically alter experimental results. Selection criteria are tailormade to each experimental conditions, which is, as the authors note, a necessity. It therefore makes little sense to blindly apply selection criteria used in one study to another, as the authors do here. This strongly limits the relevance of the conclusions of the current study.

STATISTICS

The authors do not perform statistical comparisons across studies.

COMMENTS

Large data sets of calcium imaging require an automated and standardized approach for selecting and characterizing neuronal responses. Unfortunately, there is currently no standardized method for selecting stimulus-responsive neurons across labs, with each study developing its own metric for assessing responsivity. In this study, the authors examine how different selection criteria impact measures of neuronal response properties. Using a dataset of ∼10 000 neurons from the Allen Brain Observatory, they assess how applying selection criteria previously used in five published studies impact the characterization of neuronal response properties. They find that different selection criteria select for distinct but overlapping subsets of neurons that differ in their mean preferred temporal frequency and direction selectivity (as measured by the direction selectivity index) , but have similar orientation selectivity, as measured by the circular variance method. The authors argue that differences in selection criteria may underlie differences in response properties reported across the literature. Whilst the findings of this study are not unexpected, it is to my knowledge the first to explicitly shed light on the possible caveats associated with different selection criteria applied to calcium imaging data. Consequently, this study does, in principle, stand to be of benefit to the scientific community. However, unfortunately, there are major concerns that compromise the merit of the study and its conclusions.

The main issue is that studies 1-5 not only differ in selection criteria, but as the authors note themselves, in a number of other experimental variables that could critically alter experimental results. Selection criteria are therefore tailormade to each experimental study, which is, as the authors note, a necessity. It therefore makes little sense to blindly apply selection criteria used in one study to another, as the authors do here. This strongly limits the relevance of the conclusions of the current study.

Major concerns

1) A critical flaw lies in applying different selection criteria from Studies 1-5 that were developed under vastly different experimental conditions to a common dataset. These selection criteria were developed in studies that differed in their use of anaesthesia, the types of stimuli shown, and most importantly, the calcium indicator used. Such differences likely alter the magnitude and reliability of neuronal responses, which in turn, impact the choice of selection criteria used in each study to select for responsive neurons. Critically, even though selection criteria differ across studies they may still be selecting the same kinds of neurons within their respective data set. For example, if a selectivity criteria utilizes a minimum response threshold (as Study 1, 4 and 5 do) to define responsivity, then the actual threshold set should depend on the mean response magnitudes obtained in the study. The use of the highly sensitive GCaMP6s, for example, would enable the detection of sparse activity neurons that would otherwise be undetected by indicators of lower sensitivity. Consequently, it would appear reasonable to user a higher response threshold for GCaMP6s as compared to less-sensitive indicators. Indeed, Study 1, which uses GCaMP6s, uses a responsiveness criteria of >10% mean dF/F whilst Study 5, which uses the less sensitive indicator Fura-2, uses a less strict criteria of >5% max dF/F. Despite these differences both studies find 40-50% of neurons to be responsive based on their individual criteria, which makes sense given that each criteria is adapted for the experimental conditions to which it pertains. Selection criteria are therefore tailormade to each experimental study.

This argument is not restricted to just the application of the thresholded selection criteria of Study 1 and 5, but all of the selection criteria from Studies 1-5. In each case, when the authors apply the selection criteria of each of the study to their dataset, they obtain a very different response fraction than that obtained by the original studies for which the selection criteria were specifically designed.

2) The authors suggest that differences in selection criteria may underlie differences in findings across studies, but their study offers no convincing proof for this. Studies 1-5 not only differ in selection criteria, but also in a number of other experimental variables that could critically alter experimental results. Of particular note is that the stimuli used in these and other studies greatly differ in size (full field vs patch), spatial and temporal frequencies, and pattern (pure sine-wave vs square) which are known to affect neuronal response properties and preferences. Spatial frequencies for example impact orientation/direction selectivity and preferences, the extent to which depends on whether sine or square gratings are used (Ayzenshtat et al., 2016; Jagruti et al., 2018) . Moreover, since neurons have speed preference (Andermann et al., 2011), the apparent preference for temporal frequency will depend on the spatial frequency of the stimulus (and vice versa). Thus it remains unclear to what extent selection criteria is playing a role in reported experimental differences.

3) The authors also show how neuronal response patterns change with a CV-based selection criteria with varied inclusion thresholds. This is a far more informative approach since the selection criteria is systematically varied within the dataset enabling differences in analysis to be directly compared. However, this analysis does not help to understand if possible differences in experimental findings across studies arises due to differences in the mean CV of neurons being selected. It would be far more useful if the authors could examine if the mean CV of neurons being selected in other studies (ie Studies 1-5) does indeed correlate with reported response properties in a predictable way. This would also enable them to assess how and why different selection criteria, by biasing selection towards high or low CV neurons, may impact analysis of neuronal properties.

REVIEWER 2

ADVANCES THE FIELD

This paper provides "checks" on the reliability of results from previous studies and clarifies apparently contradictory results.

STATISTICS

While it is customary to report OSI and DSI values without cross-validation, as done here, this introduces statistical biases which the authors themselves acknowledge in the paper: neurons with the worst signals end up having the highest apparent DSI. While that replicates the (incorrect) analyses from previous studies, it points to an additional cautionary tale that would be good to spell out and treat correctly in this paper.

COMMENTS

This paper provides a valuable contribution to the field primarily through a cautionary tale on the effect of inclusion criteria on reported tuning properties of neurons in V1. By extension, this should be a warning to all studies where tuning properties are calculated and compared. I think this paper is good, but it’s a little thin and I think there are two extra analyses in the same spirit that would give it a big boost.

1) You should include differences in stimulus paradigms between studies as potential confounds in the introduction, and you should mention the number of trials of each condition from each study. The number of trials has a big effect on the actual SNR in a study because the SNR increases with more trial-averaging. This has similar and potentially larger effects as the SNR of the calcium sensor. See Stringer et al, bioRxiv 2019 for the effect of the number of trials on decoding (it’s massive). Therefore, I propose that the authors study the effect of the number of trials on the tuning properties they report (OSI, DSI, TF).

2) For OSI and DSI, I think it is long overdue in this field for the calculations to be done in a statistically sound fashion. It is common to just take the average tuning curve, pick the largest response as the preferred stimulus, and then compute the OSI/DSI from that, but that is highly biased for a noisy neuron, because the largest response is not necessarily the true preferred stimulus of that neuron. In fact, if the data is noisy enough, all neurons will appear to have very high OSI/DSI, and the authors observe that exactly this effect governs differences in DSI between different inclusion criteria. The right way to do it is to divide the trials in half, compute the preferred stimulus on one half, and compute the OSI/DSI on the other half, using that preferred stimulus. This should be a standard cross-validation technique in the field, which the authors have a chance of introducing here as the right way of computing OSI/DSI.
